# Study protocol: cohort event monitoring for safety signal detection after vaccination with COVID-19 vaccines in Iran

**DOI:** 10.1186/s12889-022-13575-1

**Published:** 2022-06-09

**Authors:** Roqayeh Aliyari, Sepideh Mahdavi, Mostafa Enayatrad, Sajad Sahab-Negah, Sairan Nili, Mohammad Fereidooni, Parvin Mangolian shahrbabaki, Alireza Ansari-Moghaddam, Abtin Heidarzadeh, Fariba Shahraki-Sanavi, Mohammadreza Amini Moridani, Mansooreh Fateh, Hamidreza Khajeha, Zahra Emamian, Elahe Behmanesh, Hamid Sharifi, Mohammad Hassan Emamian

**Affiliations:** 1 Department of Epidemiology, School of Public Health, Shahroud University of Medical Sciences, Shahroud, Iran; 2 Clinical Research Development Unit, Bahar Hospital, Shahroud University of Medical Science, Shahroud, Iran; 3 Neuroscience Research Center, Mashhad University of Medical Sciences, Mashhad, Iran; 4 Department of Public Health, Faculty of Health, Kurdistan University of Medical Sciences, Sanandaj, Iran; 5 Cellular and Molecular Research Center, Birjand University of Medical Sciences, Birjand, Iran; 6 Department of Critical Care, Razi Faculty of Nursing and Midwifery, Kerman University of Medical Sciences, Kerman, Iran; 7 Health Promotion Research Center, Zahedan University of Medical Sciences, Zahedan, Iran; 8 School of Medicine, Guilan University of Medical Sciences, Rasht, Iran; 9 Infectious Diseases and Tropical Medicine Research Center, Zahedan University of Medical Sciences, Zahedan, Iran; 10 Vice-Chancellery for Health Affairs, Guilan University of Medical Sciences, Rasht, Iran; 11 Center for Health Related Social and Behavioral Sciences Research, Shahroud University of Medical Sciences, Shahroud, Iran; 12 Ophthalmic Epidemiology Research Center, Shahroud University of Medical Sciences, Shahroud, Iran; 13 Health Technology Incubator Center, Shahroud University of Medical Sciences, Shahroud, Iran; 14 HIV/STI Surveillance Research Center, Kerman University of Medical Sciences, Kerman, Iran

**Keywords:** Cohort profile, COVID-19 vaccine, Iran, Vaccination, Safety

## Abstract

**Background:**

New vaccines that are initially approved in clinical trials are not completely free of risks. Systematic vaccine safety surveillance is required for ensuring safety of vaccines. This study aimed to provide a protocol for safety monitoring of COVID-19 vaccines, including Sputnik V, Sinopharm (BBIBP-CorV), COVIran Barekat, and AZD1222.

**Methods:**

This is a prospective cohort study in accordance with a template provided by the World Health Organization. The target population includes citizens of seven cities in Iran who have received one of the available COVID-19 vaccines according to the national instruction on vaccination. The participants are followed for three months after they receive the second dose of the vaccine. For each type of vaccine, 30,000 people will be enrolled in the study of whom the first 1,000 participants are in the reactogenicity subgroup. The reactogenicity outcomes will be followed seven days after vaccination. Any hospitalization, COVID-19 disease, or other minor outcomes will be investigated in weekly follow-ups. The data are gathered through self-reporting of participants in a mobile application or phone calls to them. The study outcomes may be investigated for the third and fourth doses of vaccines. Other long-term outcomes may also be investigated after the expansion of the follow-up period. We have planned to complete data collection for the current objectives by the end 2022.

**Discussion:**

The results of this study will be published in different articles. A live dashboard is also available for managers and policymakers. All data will be available on reasonable requests from the corresponding author.The use of the good and comprehensive guidelines provided by WHO, along with the accurate implementation of the protocol and continuous monitoring of the staff performance are the main strengths of this study which may be very useful for policymaking about COVID-19 vaccination.

## Background

Although there have been three influenza-related pandemics in the world in the last centuries [1], and the spread of the Corona virus will not be the last epidemic in the world [2], with the emergence of the Corona virus 2 (SARS- CoV-2), as a new acute respiratory syndrome, in late 2019, the world entered a health crisis it had never experienced before [3]. With the World Health Organization (WHO) declaring COVID-19 disease as an epidemic on March 11, 2020, joint international efforts to develop a vaccine were intensified [4]. The vaccines, as one of the best interventions developed to control the COVID-19 pandemic, have saved countless lives [5]. However, evaluation of the performance of COVID-19 vaccines in the real world has always been necessary to understand the risks and benefits of vaccination programs, especially regarding the acceptance of the vaccines by the general public, as well as health care providers [6, 7]. Under such circumstances, local health-oriented researches increased with calls from policymakers, officials, and health executives [8]. Numerous studies have examined the efficacy and effectiveness of the COVID-19 vaccines, as well as their short- and long-term side effects [5, 9–11].

To estimate the adverse events due to the vaccines, the WHO recommended designing and implementing prospective cohort studies. Such studies can follow up vaccinated individuals regularly and record the adverse events. Publishing study protocols, as well as informing the scientific community of the studies that have been done or are being done, helps to prevent duplication and results in better coordination of research efforts. On the other hand, making study protocols available to the public has the benefit of disseminating the most modern ideas with regard to study design and data analysis [12]. This study introduces the protocol of a national cohort study in Iran to monitor adverse events and also to detect safety signals after COVID-19 vaccination.

## Methods

### Study area and participant enrollment

**Fig. 1:**
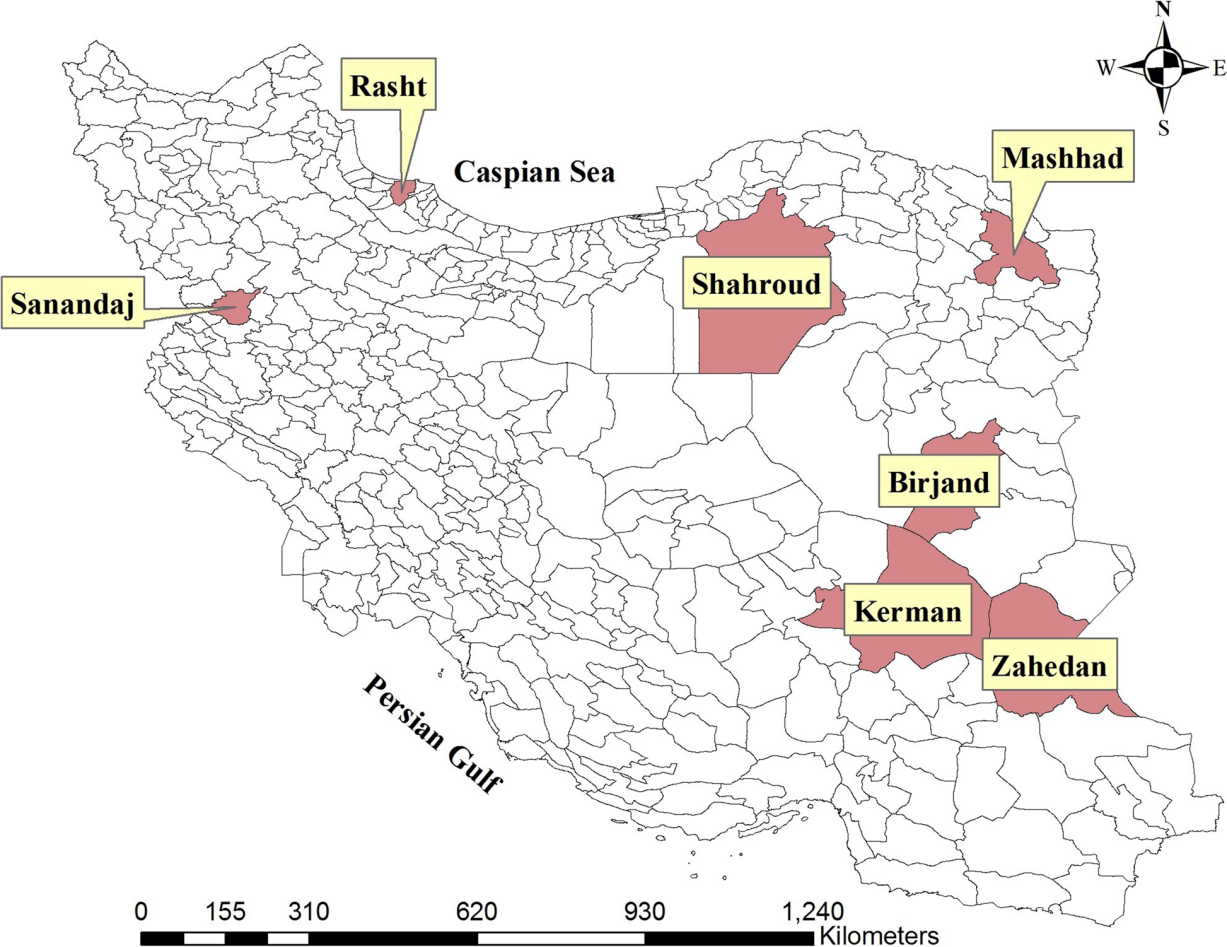
The location of study sites in Iran map (prepared by using ArcGIS software. Version 10.3. Redlands, CA: Environmental Systems Research Institute, Inc., 2014, https://www.esri.com/en-us/home)

This prospective cohort study, which is in accordance with the guidelines of the WHO [13] follows people who have received one of the COVID-19 vaccines in seven cities of Iran, namely Birjand, Kerman, Mashhad, Rasht, Sanandaj, Shahroud, and Zahedan (Fig. 1). The seven study sites were selected based on the availability of tertiary hospitals with the capacity to diagnose the AESIs, the commitment of local authorities, and the availability of eligible co-investigators around the country. Based on the vaccination program in Iran, at the beginning of the study, mainly health care workers, people over 50 years old, and those with underlying diseases were recruited. With the expansion of the vaccination program, all people over the age of five could participate in the study. Participation in this study is completely voluntary, and the recruitment is done after obtaining written informed consent from the participants. Samples of 1000 people for estimation of the incidence of local and systemic reactions and 30,000 people for other outcomes are planned to participate in each vaccine. In other words, for each vaccine, 30,000 people will be enrolled in the study, of whom the first 1,000 ones are in the reactogenicity subgroup. The Sinopharm vaccine has two subgroups of 30,000 people, one for the ones over 18 and the other for those under 18 years old. The sample size was calculated according to the guideline of the WHO to detect adverse events that occur in more than one case per 10,000 vaccinated individuals [13].

### Informed consent

First, trained health personnel explain the objectives of the study and the method of implementation to the people who receive the first dose of COVID-19 vaccines. If individuals wish to participate in the study, they are given two sheets of the informed consent form. One sheet remains with the individual, and the other is archived after the participants sign it. Then, the demographic data of the participants are recorded as well as the contact information of themselves and their next of kin.

### Data gathering and follow-up survey

The vaccination data including vaccine name and vaccine batch number, vaccinator name, and date and time of vaccination, are recorded in a specialized software program. The participants are also asked about systemic signs, including fever, nausea, chills, headache, joint pain, myalgia, malaise, and fatigue from three days before vaccination [13]. The first 1000 people for each vaccine (reactogenicity subgroup) will be under active surveillance on a daily basis for seven days after the vaccination for both the first and second doses. In the case of any local adverse effects (including pain, induration, swelling, warmness, redness, bruise, and itching at the injection site) or systemic adverse effects described above, the required data are recorded into the software. The severity of each reactogenicity adverse effect is also assessed by asking questions about the extent to which that adverse effect interferes (does not interfere, somewhat interferes, and considerably interferes) with the individuals’ daily activities (Table 1).

**Table 1:** The main variables which gathered in enrolment and follow up questionnaires

Participant Information	Reactogenicity (pre-vaccination)	Reactogenicity (post-vaccination)	Follow-up questionnaire
Study Site	Fever	Injection site Pain	Medical care
Subject ID	Body temperature	Pain Severity	Hospitalization
Subject Name	Nausea	Redness	Hospitalization date
phone number	Nausea severity	Redness size	Discharge date
Name of next kin	Malaise	Swelling	Hospitalization reason
Phone number of next kin	Malaise severity	Swelling size	Hospitalization diagnostics
Vaccine dose ID	Chills	Induration	Hospitalization report
Vaccination date	Chills severity	Induration size	Hospital ID
Vaccine brand	Headache	Bruise	COVID-19
Birth date	Headache severity	Bruise size	COVID-19-Test
Marital Status	Pain	Warmness	COVID-19 Symptom Onset Date
Sex	Pain severity	Warmness severity	ICU admission
Pregnancy	Muscle pain	Muscle pain	ICU admission Date
Lactation	Muscle pain Severity	Muscle pain Severity	Death
Chronic respiratory disease	Tired (fatigued)	Tired (fatigued)	Death date
Chronic heart disease	Tired Severity	Tired Severity	Reason of death
Chronic liver disease		Chills	Pregnancy
Chronic renal disease		Chills severity	Abortion
Diabetes		Headache	Menstrual irregularities
Immunocompromised / Immunosuppressed		Headache severity	
Obesity		Fever	
Allergy		Body temperature	
Prior Covid		Nausea	
Date of Prior Covid		Nausea severity	
History of reaction to vaccination		Malaise	
Weight		Malaise severity	
Height			

To gather the above data, a web application has been designed for participants to record their own reports. This application is installed on their mobile phones on the day of vaccination, and people are asked to record possible reactions for seven days after each injection of the vaccine. If the self-report is not done through the mobile application by 16:00, they are contacted by phone up to twice on the same day. If no response is recorded from the participants and their next of kin by 23:59, the follow-up of that day is recorded as loss to follow up for that day.

The trained officers (interviewers or data collectors) contact the participants on a weekly basis for three months (13 weeks) for single-dose vaccines and four months (17 weeks) for COVIran Barekat, Sinopharm (BBIBP-CorV), and Sputnik V. For the AZD1222 vaccine, the follow-up period is 25 weeks due to the longer interval between the two doses of the vaccine. In this study, any overnight hospitalization or reported death is recorded as a Serious Adverse Event (SAE). In addition to the active surveillance described above, weekly reports are also prepared through self-reporting of participants using the mobile application.

After sending the data by the participants using the mobile application, the information is read instantly in the registration system and used in the live management dashboard. If a participant records an important event, the trained officers are notified immediately and contact the person, and further follow-up is done actively.

### Patient and public involvement

Patients and the public are not involved in any way in this study.

### Classification of adverse events of vaccination

All hospitalized participants and cases with SAE and Adverse Events of Special Interest (AESIs) are investigated by the classification committees in each study site (Table 2). The members of these committees include infectious disease specialists, immunologists, epidemiologists, internal medicine specialists, cardiologists, clinical pharmacologists, and other specialists, if necessary. The committee is responsible for the examination of the medical records and the attribution of adverse events to the COVID-19 vaccines.

**Table 2:** Adverse events of special interest (AESIs) and their risk windows.^a^

No	Body system	AESI	Risk interval (Days after vaccination)
1	Cardiac	Acute cardiovascular injury	D1-D42
2	Dermatologic	Chilblain like lesions	D1-D42
3	Dermatologic	Single organ cutaneous vasculitis	D1-D42
4	Dermatologic	Erythema multiforme	D1-D42
5	Endocrine	Pancreatitis	D1-14
6	Endocrine	Subacute thyroiditis	D1-42
7	Gastrointestinal	Acute liver injury	D1-D42
8	Hematologic	Coagulation disorder (thromboembolism)	D1-D42
9	Hematologic	Thrombocytopenia	D1-D42
10	Immunologic	Vaccine-associated enhanced disease	Unknown
11	Gastrointestinal Respiratory	Anosmia, ageusia	Unknown
12	Immunologic	Anaphylaxis	D0-D7
13	Musculoskeletal	Acute aseptic arthritis	D1-D42
14	Musculoskeletal	Rhabdomyolysis	D1-D7
15	Neurologic	Acute disseminated encephalomyelitis	D1-D42
16	Neurologic	Bell’s palsy	D1-D42
17	Neurologic	Generalized convulsion	D1-D42
18	Neurologic	Guillain-Barré syndrome	D1-D42
19	Neurologic	Meningoencephalitis	D1-D42
20	Renal	Acute renal injury	D1-D42
21	Respiratory	Acute respiratory distress syndrome	D1-D42
22	Immunologic	Multisystem inflammatory syndrome in children [Only for under 18 Y/O subgroup]	D1-D42

^a^Adapted from WHO template (Reference No. [13])

If a participant becomes pregnant, the follow-up period will continue until delivery or the end of the pregnancy, and the pregnancy outcome is recorded.

### Data analysis and outcomes

Descriptive statistics, including response rate; adverse events by vaccine brand, vaccine dose, age, and sex groups; and other demographic characteristics of participants will be provided. The incidence of SAE and AESI by vaccine brand and the time passed since vaccination dates will be reported. The incidence rate of COVID-19 infection, hospitalization, and death will be provided by calculating the total person/time since the vaccination dates. By considering the calendar time as the time scale, the Cox proportional-hazards model will be used to determine factors associated with COVID-19, infection, and hospitalization and compare different vaccine brands.

The reactogenicity effects will be presented based on vaccine brands, seven days after the vaccination. These reactions will be assessed by comparing the observed rate with the expected rate (obtained from the questionnaires filled three days before the vaccination).

### Quality control, monitoring, and reporting

The required staff have been selected through interviews and trained in a four-hour workshop. The first 30 people recruited in each study site are considered in a pilot study and do not enter in the final analysis. The daily performance of the staff is monitored through direct observations and review of the data recording reports in the study software. In the case of any problems in the registration system, the problem is specified and corrected on the same day. Any secular trend is identified and followed through the live and interactive management dashboard (available at: http://shmu-ivss.ir:3838/sample-apps/IVSS).

The interim progress reports are performed weekly. The study software provides common reports. The interim analysis and related reports are provided at monthly intervals. If the recorded adverse events exceed the expected amount obtained in the clinical trial studies, the investigators immediately inform the national authorities. The final analysis and report will be prepared four weeks after the end of the study and will be provided to the national immunization managers.

## Discussion

### Findings to date

**Fig. 2:**
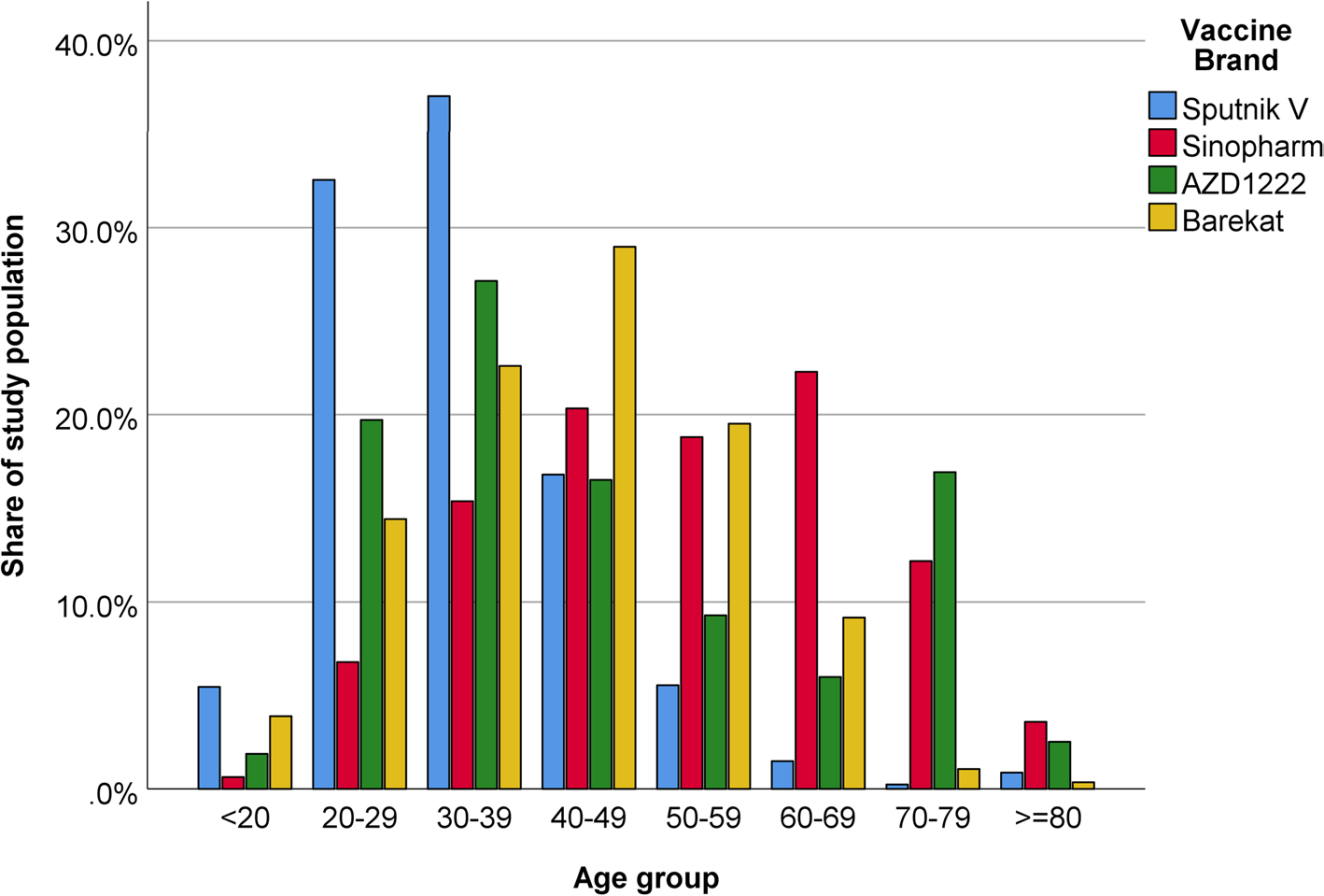
Age distribution of participants by vaccine brands

Until the last version of this protocol (May 29, 2022), 105,894 individuals have participated in the study. The mean age of participants is 40.4 ± 19.5 year (range: 5 to 105), and 54,959 (51.9%) of whom are male. Figure 2 shows the age distribution of participants. The number of participants and the percent of those who have received the second dose of vaccine (in parentheses) for each vaccine are 31,690 (96.9%) for Sinopharm, 20,195 (87.6%) for Sputnik V, 23,780 (90.0%) for AZD1222, 16,111 (51.6%) for Sinopharm in under 18 years old participants, and 14,118 (88.6%) for COVIran Barekat.

The number of incident cases of AESI, SAE, abortion, and mortality due to COVID-19 equals 12, 21, 48, and 57, respectively. The AESIs includes four cases with coagulation disorders, (three after AZD1222 and one after Sinopharm vaccination), as well as four cases of generalized convulsion after receiving Sinopharm, Sputnik, and AZD1222 (two cases) vaccines. In addition, four cases of Guillain–Barre syndrome, Diabetic ketoacidosis, Pericarditis and Sub-acute Thyroiditis were detected after receiving Sputnik V, Sinopharm, COVIran Barekat and Sputnik V respectively.

The SAEs diagnosed so far include severe allergy (two cases), abdominal pain (two cases), palpitation and cardiac arrhythmia (three cases), chest pain (five cases), high fever and agitation (two cases), vertigo and faint (three cases), and high blood pressure (two cases).

Sinopharm had higher rate of breakthrough infection than other vaccines. For hospitalization and death, all vaccines provided similar protection after 14 days from the second dose, while AZD1222 was superior to other vaccines in participants receiving only a single dose. Prior Covid-19 disease had a significant protective role on Covid-19 infection. Diabetes, respiratory, cardiac and renal diseases were comorbidities associated with a higher risk of COVID-19 infection.

After injection of the second dose of the vaccine, the incidence of local and systemic adverse effects for all four vaccines has been lower than the first dose. The most frequent local adverse effect has been the pain (in the injection site) while the most frequent systemic adverse effect has been fatigue. AZD1222 has resulted in the highest frequency of local and systemic adverse effects compared to other vaccines.

During the follow-up period, 6,681 cases of COVID-19 have been detected, about half of which have occurred before receiving the second dose. Among the participants with COVID-19, 647 patients have been hospitalized and 57 patients have died.

### Further details

The main strengths of this study include its large sample size, investigation and comparison of four vaccines, active surveillance, weekly follow-ups, and investigation and classification of all hospitalized participants. However, virus variants could not be determined, and there is no control group for investigating vaccine effectiveness which can be considered a limitation of the current study.

## Data Availability

All data of this study can be provided at the request of the corresponding author (Prof. Mohammad Hassan Emamian, via emamian@shmu.ac.ir). All researchers around the world can send their proposed titles. After screening in a scientific committee, the new titles will be approved and the required data will be available for researchers. The new articles and reports then will be prepared by collaboration with researchers of this study.

## Abbreviations

WHO: World Health Organization
AESI: Adverse Events of Special Interest
SAE: Severe Adverse Event
